# A SAP30 Complex Inhibits IFN-β Expression in Rift Valley Fever Virus Infected Cells

**DOI:** 10.1371/journal.ppat.0040013

**Published:** 2008-01-25

**Authors:** Nicolas Le May, Zeyni Mansuroglu, Psylvia Léger, Thibaut Josse, Guillaume Blot, Agnès Billecocq, Ramon Flick, Yves Jacob, Eliette Bonnefoy, Michèle Bouloy

**Affiliations:** 1 Unité de Génétique Moléculaire des Bunyavirus, Institut Pasteur, Paris, France; 2 Régulation de la Transcription et Maladies Génétiques, CNRS UPR2228, UFR Biomédicale, Université Paris Descartes, Paris, France; 3 BioProtection Systems Corporation, Ames, Iowa, United States of America; 4 Unité de Génétique, Papillomavirus et Cancer Humain, Institut Pasteur, Paris, France; Mount Sinai School of Medicine, United States of America

## Abstract

Rift Valley fever virus (RVFV) nonstructural protein NSs acts as the major determinant of virulence by antagonizing interferon β (IFN-β) gene expression. We demonstrate here that NSs interacts with the host protein SAP30, which belongs to Sin3A/NCoR/HDACs repressor complexes and interacts with the transcription factor YY1 that regulates IFN-β gene expression. Using confocal microscopy and chromatin immunoprecipitation, we show that SAP30, YY1, and Sin3A-associated corepressor factors strongly colocalize with nuclear NSs filaments and that NSs, SAP30 and Sin3A-associated factors are recruited on the IFN-β promoter through YY1, inhibiting CBP recruitment, histone acetylation, and transcriptional activation. To ascertain the role of SAP30, we produced, by reverse genetics, a recombinant RVFV in which the interacting domain in NSs was deleted. The virus was unable to inhibit the IFN response and was avirulent for mice. We discuss here the strategy developed by the highly pathogenic RVFV to evade the host antiviral response, affecting nuclear organization and IFN-β promoter chromatin structure.

## Author Summary

Rift Valley fever is a viral mosquito-borne disease affecting ruminants and humans. The disease occurs in Africa and recently it spread to the Arabian Peninsula. In humans, infection can progress to fatal hemorrhagic fever and in ruminants it leads to hepatitis, abortions, or deaths of young lambs. It has been previously shown that the RVFV protein NSs is the major factor of virulence and that pathogenicity is associated with the lack of interferon production. In this study, we analyzed the interaction of NSs with SAP30, a subunit of complexes intervening in gene transcription regulation. We show that SAP30 through its binding to NSs on one hand and to YY1 (the activator/repressor of interferon transcription) on the other hand, forms a multiprotein repression complex on the interferon β promoter. As a consequence, interferon expression is blocked, allowing virus to invade the whole organism. The relevance of the NSs–SAP30 interaction was ascertained by constructing a recombinant virus in which the interacting domain is disrupted. This virus is able to induce interferon expression and when inoculated to the mouse model it was found nonpathogenic.

## Introduction

It is now well established that in eukaryotic cells, transcriptional activation of finely regulated inducible genes requires disruption of chromatin structure in order to allow the access of RNA polymerase to DNA (for recent reviews see [1–3]). The nucleosome is the basic unit of chromatin, consisting of DNA wrapped around an octamer of histones (two of each H2A, H2B, H3 and H4). This organized structure is a highly dynamic molecular edifice whose remodeling occurs in response to internal and external signals through post-translational modifications of histones, such as acetylation and methylation, as well as ATP-dependent nucleosome reorganization carried out by different types of multiprotein complexes. Promoter recruitment of chromatin remodeling complexes usually relies on transcription factors that bind to a specific DNA sequence and establish protein-protein interactions with chromatin remodeling complexes.

The interferon β (IFN-β) gene is a well characterized example of these regulatory mechanisms. While the IFN-β gene is constitutively repressed in non-infected cells, it is normally turned on as soon as a virus infects cells, establishing an antiviral state [4–6]. Activation of the IFN-β gene is transient since it undergoes a rapid post-induction turnoff between 10 and 12 h after infection [7,8]. Transcriptional regulation of the IFN-β promoter requires specific binding of transcription factors as well as the orderly recruitment of chromatin remodeling complexes on the promoter region [9,10]. In the absence of virus infection, an until now non-identified corepressor complex maintains deacetylated lysine residues of histone H3 and H4 positioned on the IFN-β promoter region [11,12]. Shortly after infection, the enhanceosome consisting of NF-kB, IRFs, ATF2/cJun and the architectural protein HGMI(Y) is assembled at the Virus Responsive Element (VRE) region. The enhanceosome instructs a program of chromatin modifying activities by recruiting histone acetyltransferases CBP/p300 and Gcn5/PCAF that acetylate lysine residues of histones H3 and H4, especially K8H4 and K14H3, leading to the recruitment of Pol II holoenzyme and the Swi-Snf nucleosome remodeling complex [9,13]. More recent work has identified transcription factor YY1 as an important factor during IFN-β transcriptional regulation, intervening both as a HDAC-dependent repressor and as an activator essential to allow virus-induced CBP-recruitment and K8H4/K14H3 acetylation on the promoter region after virus infection [14–16].

Rift Valley Fever Virus (RVFV) is an arthropod-borne virus circulating in sub-Saharan Africa, Egypt and Arabic Peninsula, transmitted mostly by Aedes sp. and *Culex* mosquitoes. RVFV infection can lead to fatal hepatitis with hemorrhagic fever in humans and to high mortality rates in ruminants [17,18]. The virus belongs to the Bunyaviridae family (genus *Phlebovirus*), a family of spherical enveloped viruses that possess a single stranded segmented RNA genome composed of a large (L), a medium (M) and a small (S) segment [19,20]. The L and M segments are of negative polarity and code respectively for the L RNA-dependent polymerase and the glycoprotein precursor, whereas the S segment utilizes an ambisense strategy and codes for the nucleoprotein N and the nonstructural protein NSs [21]. Although all the steps of the viral cycle occur in the cytoplasm, NSs (31 kDa, 265 amino-acids) forms filamentous structures in the nucleus of infected cells [22–24]. Besides being a general inhibitor of cellular RNA synthesis by interacting with p44 subunit of TFIIH [25], this viral protein strongly antagonizes IFN-β production shortly after infection [26–28].

Here, we demonstrate using the yeast two hybrid system, biochemical methods and confocal microscopy that RVFV NSs protein interacts with SAP30 (Sin3A Associated Protein 30) which is a subunit of Sin3A corepressor complexes [29,30] as well as a partner of YY1 [31]. Confocal microscopy demonstrates that in RVFV-infected cells, the virus induces a strong subnuclear redistribution of SAP30, YY1 and Sin3A-associated corepressor factors that colocalize with the nuclear NSs filaments. Chromatin immunoprecipitation (ChIP) experiments showed that in RVFV infected cells the IFN-β promoter is maintained in a transcriptionally silent state, interacting with NSs and SAP30 through YY1. Moreover, a RVFV mutant produced by reverse genetics which lacks the SAP30 interacting domain, was unable to inhibit IFN-ß production and was avirulent in mice. We discuss here the importance of RVFV-induced subnuclear redistribution of chromatin-remodeling corepressor components which are trapped into a nuclear filamentous structure for targeting IFN-β gene into a repressed environment and blocking the antiviral response of host cells.

## Results

### RVFV Non Structural Protein NSs Interacts with SAP30

**Figure 1:**
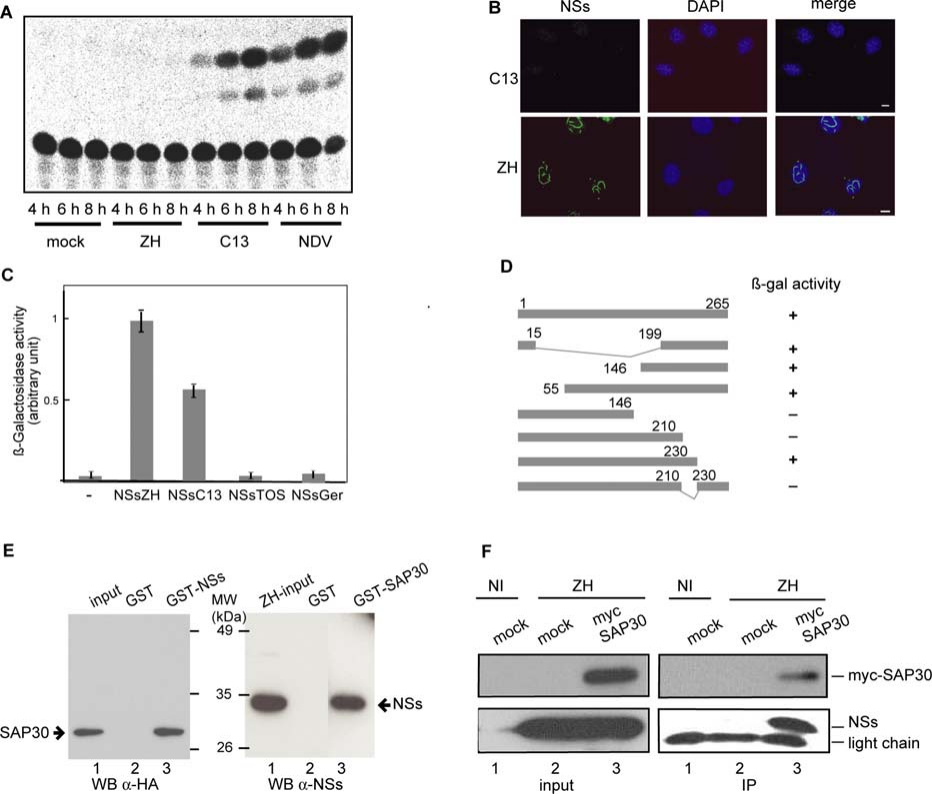
ZH NSs Protein Inhibits IFN-β Promoter and Interacts with Host Protein SAP30 (A) L929 wt330 cells, carrying an integrated wild type muIFN-β promoter fused to CAT reporter gene, were mock infected or infected by RVFV ZH or C13 or with NDV. Total cell extracts were prepared at 4, 6 and 8 h p.i. and CAT actvity was measured. (B) Non-confocal conventional fluorescence microscopy was used to analyze the nuclear distribution of NSs filaments in murine L929 cells infected by C13 or ZH. Presence of NSs filament detected using rabbit polyclonal anti-NSs antibody (green) or total DNA distribution revealed with Hoechst 33258 are shown respectively, in left and middle panels. Merged images are shown in right panels. Scale bars, 10 μm. (C) For yeast two-hybrid screening, AH109 yeast were co-transformed by pACT2-SAP30_1–152_ that expressed Gal4 transactivating domain fused to the open reading frames corresponding to the 152 first aa of SAP30 and pGBKT7, pGBKT7-NSs_ZH_, pGBKT7-NSs_C13_, pGBKT7-NSs_TOS_, or pGBKT7-NSs_GER_ in which NSs from RVFV ZH or C13 or NSs proteins from Toscana (TOSV) and Germiston (GERV) bunyaviruses were fused to the Gal4 DNA-binding domain. The values of β galactosidase activity represent at least four independent experiments with SD bars. (D) Two-hybrid system using full length wild type NSs_ZH_ or the deleted forms. The numbers indicate the amino acid position in the reference sequence. The sequence lacking amino acids 16–198 correspond to C13. (E) GST-NSs (left panel) or GST-SAP30 (right panel) was incubated with an extract from 293 cells transfected with the HA tagged-SAP30 expressing plasmid (left panel) or from ZH infected L929 cells (right panel). After extensive washing, the proteins bound to the beads were analysed by western blots using antibodies against HA (left panel) or NSs (right panel). The Coomassie blue staining showing that equivalent amounts of GST fusion proteins were loaded on the beads is not shown. (F) HEK 293 cells were transfected with either pCS2-Myc (lanes 1,2) or pCS2-Myc-SAP30 (lane 3) and either not infected (lane 1) or infected with ZH (lanes 2,3). Cell lysates were precipitated with anti-myc (9E10) antibody. Crude lysates (input) and the precipitated proteins (IP) were detected with anti-myc and anti-NSs antibodies.

We have previously shown that NSs inhibits IFN-β expression immediately after infection with the virulent RVFV strain ZH548 (ZH), without inhibiting IFN-β-specific transcription factors such as IRF3, NF-κB and ATF2 that are normally activated and translocated to the nucleus in RVFV-infected cells [26]. In an attempt to decipher the mechanism developed by RVFV to inhibit IFN-β gene expression, we assessed the specific effect of RVFV infection on IFN-β gene expression by infecting the previously described murine fibroblastic L929 wt330 cell line carrying a stably integrated wild-type muIFN-β promoter (from position −330 to +20) CAT reporter construct upon which chromatin structure has been fully reconstituted [16]. These cells were either non-infected or infected with the virulent ZH [32] which has a fully active NSs protein or with the non-virulent RVFV strain Clone 13 (C13) that produces an unstable truncated NSs protein Δ16–198 rapidly degraded by the proteasome pathway [28,33]. As expected, no CAT activity was detected either in uninfected cells or in cells infected with the virulent strain ZH whereas in agreement with our previously published results, a high level of activity, increasing with time post infection, was observed in C13-infected cells (Figure 1A). This activity was similar to the one observed in Newcastle Disease Virus (NDV)-infected cells used here as a positive control. The ZH NSs protein formed a filamentous structure in the nuclei of murine L929-infected cells (Figure 1B) equivalent to the one previously described in several other human and animal cells [22,23,25].

Using the previously described [25] yeast two hybrid system with NSs_ZH_ as a bait to screen a cDNA library from mouse embryo, we identified SAP30 (Sin3A Associated protein 30, Swiss Prot accession number 088574) as a partner of NSs (Figure 1C). A possible NSs-SAP30 interaction appeared quite relevant with respect to IFN-β gene inhibition since SAP30 is a subunit of several corepressor complexes associated to N-CoR and/or Sin3 [29,30] as well as a direct partner of transcription factor YY1 [31] that directly interacts and regulates IFN-β gene expression. To assess the specificity of the interaction of SAP30 with NSs, yeast cells were co-transformed with pACT2-SAP30 together with pGBKT7 or pGBKT7-NSs_RVFVZH_, -NSs_TOSV_, -NSs_GERV_- that express the NSs proteins from RVFV, Toscana and Germiston bunyaviruses, respectively. A specific interaction between SAP30 and RVFV NSs was confirmed whereas NSs_TOSV_, NSs_GERV_ did not interact with SAP30 (Figure 1C). In this assay, the truncated NSs protein from C13 (NSs_C13_) also interacted with SAP30 indicating that the interaction between SAP30 and NSs did not require amino acids 16–198 of NSs which are absent in NSs_C13_ (Figure 1D). Indeed, the data reported in Figure 1D clearly showed that the interacting domain in NSs was located at the COOH terminal region, between amino acids 210 to 230. It should be noted that although C13 NSs interacts with SAP30 in yeast because it is stabilized as a fusion protein, it is non functional in C13 infected cells since it is degraded by the proteasome [28], allowing to use C13 infections as negative controls.

The NSs-SAP30 interaction was further confirmed using GST pull-down assay. After purification on glutathione Sepharose beads, the NSs protein expressed as a GST fusion protein was incubated with extracts of cells expressing a full length HA tagged-SAP30 protein after transfection with plasmid pCi-HA-SAP30. The HA tagged SAP30 protein was found to be associated with GST-NSs but not with GST alone used here as a negative control (Figure 1E, left panel). The reciprocal experiment was also carried out, using GST-SAP30 protein incubated with extracts from ZH infected cells. As shown in Figure 1E (right panel), NSs was found to bind to GST-SAP30 but not to GST alone.

To further analyze the NSs-SAP30 interaction in vivo when NSs is organized as a filamentous structure, we set up immunoprecipitation experiments from nuclear extracts of ZH-infected cells expressing transiently myc-tagged SAP30. Immunoprecipitation with anti-myc antibodies not only pulled down myc-tagged SAP30 but also the viral NSs present in the complex (Figure 1F).

###  SAP30 and YY1 Strongly Colocalize with the NSs Filament

**Figure 2:**
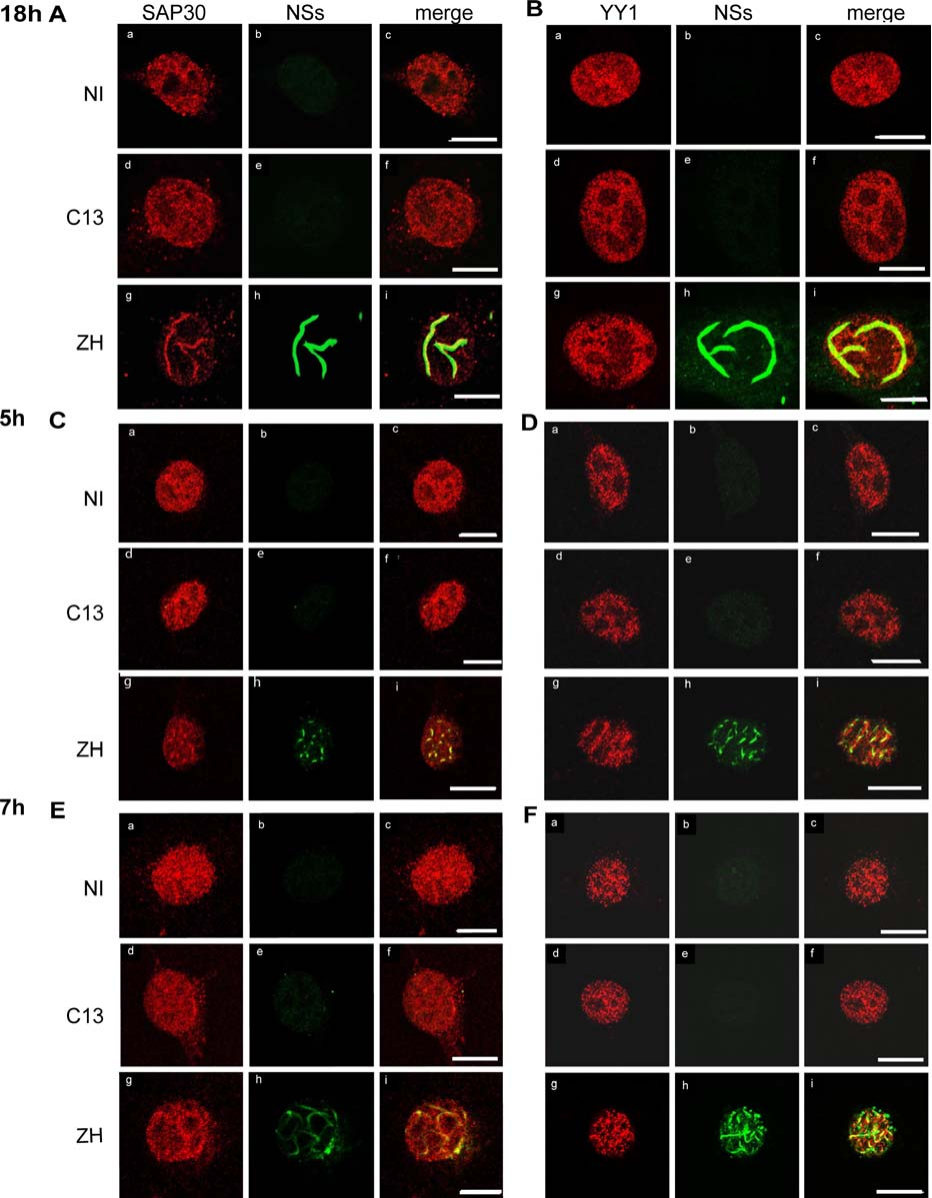
Endogenous SAP30 and YY1 Colocalize with NSs Filaments in the Nuclei of ZH Infected Cells Colocalization of endogenous SAP30 (A, C, and E) and YY1 proteins (B, D, and F) with NSs filament was analyzed by confocal microscopy in L929 wt330 cells uninfected (NI) or infected with C13 or ZH at m.o.i. 5 collected at 18 h p.i. (A and B), 5h (C and D) or 7 h p.i. (E and F). Each row represents a single optical section of the same nucleus. A, C, and E) Left panels (a, d, g) correspond to SAP30 distribution revealed with goat polyclonal anti-SAP30 antibodies. Middle panels (b, e, h) show subnuclear NSs distribution detected with anti-NSs rabbit polyclonal antibodies. Merged images of SAP30 and NSs are shown on right panels (c, f, i). (B, D, and F) Left panels (a, d, g) correspond to YY1 distribution revealed with mouse monoclonal anti-YY1 antibody. Middle panels (b, e, h) show subnuclear NSs distribution detected with anti-NSs rabbit polyclonal antibody. Merged images of YY1 and NSs are shown on right panels (c, f, i). Scale bar, 10 μm.

A recent study demonstrated that SAP30 interacts directly with YY1 [31], a transcription factor involved in the regulation of expression of numerous genes [34] including IFN-β [14–16,35]. Since nuclei of cells infected by ZH RVFV exhibit characteristic filaments containing NSs [22,23], it was of interest to determine whether SAP30 and YY1 colocalized with NSs filaments. In non-infected and C13-infected cells, host protein SAP30 appeared homogeneously distributed in the nucleoplasm, with the exception of nucleoli (Figure 2A, 2C, 2E a-f). In contrast, an important change in the subnuclear distribution of SAP30 was observed at 18 h after ZH infection so that SAP30 appeared predominantly colocalizing with the NSs filament (Figure 2A g-i) with almost all of SAP30 included into the NSs filament.

Like SAP30, YY1 was also present in the nuclei of NI and C13-infected cells (Figure 2B, 2D, 2F a-f). However, YY1 appeared less homogeneously distributed than SAP30, being apparently excluded from large zones of the nucleoplasm. As observed in Figure 2B (g-i) at 18 h after ZH infection, the NSs filament appeared clearly located in zones of higher YY1 concentration. (Figure 2B g-i). Images shown in Figure 2A and 2B represent cells analyzed at 18 h post infection when the NSs filament is fully formed. Viral NSs protein is produced shortly after infection [36] and can be detected in the nucleus as early as 3–5 h p.i. [25]. Interestingly, colocalization of SAP30 and YY1 with NSs could be observed as early as 5 and 7 h p.i. (Figure 2C–2F g-i) even before the NSs filament was completely formed. At these early times after infection, NSs colocalized perfectly with SAP30 whereas colocalization with YY1 appeared only partial.

### A Complex Containing NSs and SAP30 Is Recruited on the IFN-β Promoter via YY1 after ZH Infection

**Figure 3:**
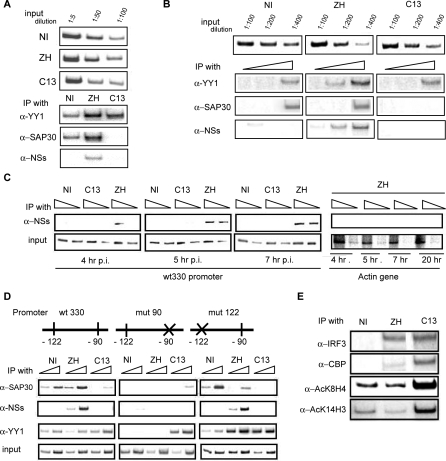
A NSs/SAP30/YY1 Complex Is Recruited on the Silent IFN-β Promoter through YY1 −90 Site L929 cells (A) or L929 wt330 (B, C, and E), or L929 wt330, mut90, and mut122 (D) were infected with ZH or C13 at a m.o.i. of 5 and collected at 6 h p.i. or as indicated (C). Input and DNA immunoprecipitated (IP) with specific antibodies as indicated, were amplified with primers specific for the endogenous IFN-β promoter (A), for the integrated murine wild type wt330 IFN-β promoter (B-E), for the mutated integrated promoters (D) or for the murine β-actin gene (C). Schematic representation of murine IFN-β promoters either wild type (wt330) or mutated at the YY1 binding site present at position −90 (mut90) or −122 (mut122) is shown in D. Inputs are shown as controls except in E where they are the same as in D (wt330). Triangles indicate increasing amounts of DNA used during PCR reactions and corresponding in (B) to 1 μl, 2 μl, or 3 μl of 1:5 dilution of DNA immunoprecipitated with a-YY1 and a-SAP30 and of 1:50 dilution of DNA immunoprecipitated with a-NSs; (C) 1 μl and 2 μl of 1:50 dilution of DNA immunoprecipitated with a-NSs and 1 μl of 1:1,000 and 1:200 dilution of input DNA; (D) 1 μl of 1:5 and 1:1 dilution of DNA immunoprecipitated with a-YY1 and a-SAP30, 1 μl and 2 μl of 1:50 dilution of DNA immunoprecipitated with a-NSs and 1 μl of 1:1,000 and 1:200 dilution of input DNA.

Since YY1 has been previously shown to directly interact with the IFN-β promoter, we carried out ChIP experiments in order to analyze the eventual recruitment of a NSs/SAP30/YY1 complex on the promoter after ZH-infection. Genomic DNA from non-infected and ZH- or C13-infected L929 and L929 wt330 cells was immunoprecipitated with antibodies directed against YY1, SAP30 and NSs. The amount of precipitated IFN-β promoter DNA was determined by PCR using primers specific for either the endogenous IFN-β promoter present in L929 cells (Figure 3A) or the integrated wild type promoter present in L929 wt330 cells (Figure 3B). Analysis of the endogenous promoter shown in Figure 3A indicated that YY1 binds to the promoter in non-infected as well as in ZH- and C13-infected cells. Contrary to YY1, SAP30 was associated to the IFN-β promoter only in non-infected and ZH-infected cells when the promoter is maintained transcriptionally silent. The interaction of SAP30 with the IFN-β promoter was disrupted after C13 infection when the promoter was activated. As expected, no interaction between NSs and the IFN-β promoter was observed in NI and C13-infected cells whereas a reproducible interaction of NSs with the promoter was observed after ZH infection. The same results were obtained after immunoprecipitation of the integrated wt330 IFN-β promoter present in L929 wt330 cells (Figure 3B) indicating that, as expected from our previous work, the integrated wild type wt330 promoter behaved as the endogenous (wild type) promoter. These experiments were carried out with cells harvested at 6 h p.i. but Figure 3C shows that NSs was found to interact with the IFN-β promoter as early as 4 and 5 h after ZH infection. This interaction seems specific since no amplification of the murine β-actin gene was obtained from the NSs immunoprecipitate either at 4, 5, 7 or 20 h p.i (Figure 3C, actin gene).

To investigate if the recruitment of the NSs/SAP30 complex on the IFN-β promoter required the presence of functional YY1 binding sites, we made use of the mutated L929 cell lines mut90 and mut122 that contain a stably integrated muIFN-β promoter (from position −330 to +20) mutated at either the −90 (mut90 promoter) or the −122 (mut122 promoter) YY1 binding sites. In both these promoters, only one base was mutated, present in the respective YY1 core binding motifs essential to allow YY1 binding at the corresponding site [15,16]. When these cells were non-infected or infected with C13 or ZH-RVFV strain and analyzed by ChIP assays (Figure 3D), we found that neither the anti-NSs nor the anti-SAP30 antibodies immunoprecipitated the mut90 IFN-β promoter whereas both these antibodies immunoprecipitated the mut122 promoter with a pattern similar to the one observed for the wild type wt330 promoter. These results strongly suggested that a functional YY1 binding −90 site was required for NSs and SAP30 to interact with the IFN-β promoter.

### ZH Infection Inhibits YY1 Binding to Its −122 Site, CBP Recruitment and Histone Acetylation on the IFN-β Promoter

Simultaneous binding of YY1 to both its −90 and −122 sites has been previously described as necessary to allow virus-induced CBP-recruitment, histone acetylation and correct activation of the promoter [16]. As shown in Figure 3D (anti-YY1 lane), binding of YY1 to its −122 site (mut90 promoter), that was induced after C13 infection and is required to allow CBP promoter recruitment, was completely inhibited after ZH infection (see mut90 promoter).

Activation of the IFN-β promoter requires IRF3 and CBP recruitment as well as K8H4 and K14H3 acetylation [9,13]. As expected, no binding of IRF3 or CBP was observed on the IFN-β promoter isolated from constitutively repressed uninfected cells (Figure 3E). Binding of IRF3 to the promoter was observed after C13 as well as after ZH infection, indicating that IRF3 is not only activated [26] but also recruited in vivo on the IFN-β promoter after ZH infection (Figure 3E). Contrary to IRF3, recruitment of CBP normally observed after C13 infection was inhibited after ZH infection. Inhibition of CBP recruitment was accompanied by the subsequent inhibition of K8H4 and K14H3 acetylation on the promoter region after ZH infection (Figure 3E).

In Figure 3E, it can be noted that K8H4 and K14H3 were not acetylated after ZH infection and that AcK14H3 especially, was deacetylated. Since SAP30 has been described as participating in the formation of corepressor complexes containing HDACs 1, 2 and/or 3, we postulated that repression of the IFN-β gene expression after ZH infection could be the result of two events: i) absence of CBP recruitment and histone acetylation probably related to the inability to recruit YY1 to its −122 site and ii) stabilization of already present or *de novo* recruitment of corepressor complexes containing HDAC activities on the IFN-β promoter region.

### SAP30-Associated Corepressor Complexes Colocalize with the NSs Filament and Interact with the IFN-β Promoter after ZH Infection

**Figure 4:**
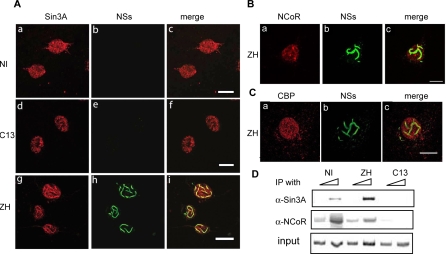
Sin3A and N-CoR, but Not Co-Activator CBP, Interact with IFN-β Promoter in ZH Infected Cells Colocalization of endogenous Sin3A (A), N-CoR (B) and CBP proteins (C) with NSs filament was analyzed by confocal microscopy in L929 wt330 cells uninfected (NI) or infected with ZH or C13 at m.o.i. 5 collected at 18 h p.i. Each row represents a single optical section of the same nucleus. (A) Left panels (a, d, g) correspond to Sin3A distribution revealed with rabbit anti-Sin3A polyclonal antibody. Middle panels (b, e, h) show subnuclear NSs distribution detected with mouse anti-NSs polyclonal antibody. Merged images of Sin3A and NSs are shown on right panels (c, f, i). (B and C) Left panel (a) corresponds to NCoR (B) or CBP (C) distribution revealed with goat anti-NCoR polyclonal antibody or rabbit anti-CBP polyclonal antibody. Middle panel (b) shows subnuclear NSs distribution detected with rabbit anti-NSs polyclonal antibody. Merged images are shown in right panels (c). Scale bar, 10 μm. (D) Inputs or DNA immunoprecipitated (IP) with anti-Sin3A and anti-NCoR antibodies collected from murine L929 wt330 cells either uninfected (NI) or 6 h after infection by ZH or C13 was amplified with specific primers.

Transcription factor YY1 is able to interact with HDACs 1, 2 or 3 either directly or indirectly [37], indirect interaction taking place probably *via* SAP30 [31]. Besides, SAP30 is part of multiprotein repressor complexes that may comprise Sin3A and/or NCoR which themselves interact directly with either HDACs 1 and 2 (Sin3A) or HDAC 3 (NCor). Using confocal microscopy, we analyzed the subnuclear distribution of Sin3A and NCoR in murine L929 cells either non-infected or after infection by C13 or ZH. As shown in Figure 4A, the subnuclear distribution of Sin3A observed in non-infected (a, c) and C13-infected (d, f) cells was affected after ZH-infection (g, i), Sin3A colocalizing with the NSs filament in a way similar to what we have previously observed in the case of SAP30. In contrast, NCoR colocalized only partially with the NSs filament (Figure 4B a-c and Figure S1) and no colocalization of co-activator CBP with the NSs filament was observed (Figure 4C a-c and Figure S1).

As shown in Figure 4D, NCoR appeared associated to the IFN-β promoter before virus infection when the promoter is in a constitutively silent state, and at this stage the presence of Sin3A on the IFN-β promoter was only weakly detected. In agreement with immunofluorescence results, binding of Sin3A but not NCoR to the IFN-β promoter was enhanced after ZH infection. Nevertheless, NCoR remained bound to the IFN-β promoter in ZH-infected cells while both Sin3A and NCoR were released from the promoter after C13 infection during promoter transcriptional activation (Figure 4D).

**Figure 5:**
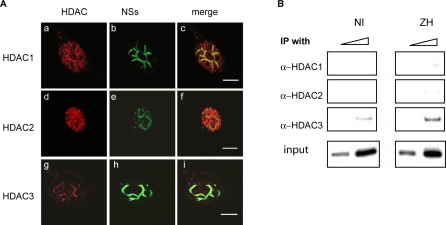
Recruitment of HDAC-3 on the IFN-β Promoter Colocalization of endogenous HDACs-1, 2, and 3 with NSs filament was analyzed by immunofluorescence technique and confocal microscopy in L929 wt330 cells uninfected (NI) or infected with ZH or C13 at m.o.i. 5 collected at 18 h p.i. Each row represents a single optical section of the same nucleus. (A) Left panels (a, d, g) correspond to HDAC-1, 2, or 3 subnuclear distribution revealed with mouse anti-HDAC1, 2, or 3 monoclonal antibodies. Middle panels (b, e, h) show subnuclear NSs distribution detected with rabbit polyclonal anti-NSs antibody. Merged images of HDACs-1, 2, or 3 and NSs are shown on right panels (c, f, i). Scale bar, 10 μm. (B) Inputs and DNA immunoprecipitated (IP) with anti-HDACs-1, 2, and 3 antibodies collected from murine L929 wt330 cells either uninfected (NI) or 6 h after infection by ZH was amplified with specific primers.

Confocal microscopy was also used to analyze the subnuclear distribution of HDACs 1, 2 and 3, that have been described to interact with SAP30/Sin3A/NCoR complexes (Figure 5A and Figure S2). Even though HDAC-1 and 2 were not excluded from the filament, neither HDAC-1 nor HDAC-2 completely colocalized with the NSs filament. Whereas HDAC-1 (Figure 5A a-c, Figure S2A) only partially colocalized with the NSs filament, no specific colocalization of HDAC-2 with the filament was observed (Figure 5A d-f, Figure S2B). Contrary to HDACs 1 and 2, almost all HDAC-3 colocalized with the NSs filament (Figure 5A g-i, Figure S2C).

ChIP experiments were carried out on the wt330 IFN-β promoter with anti-HDAC 1, 2 and 3 antibodies in non-infected cells as well as in ZH-infected cells (Figure 5B). Despite the weakness of the signal obtained, the interaction of HDAC 3 with the IFN-β promoter that was detected in non-infected cells was reproducibly found to be enhanced after ZH infection (Figure 5B).

### Disruption of NSs-SAP30 Interaction Is Correlated with the Loss of the Capacity to Inhibit IFN-β Expression

**Figure 6:**
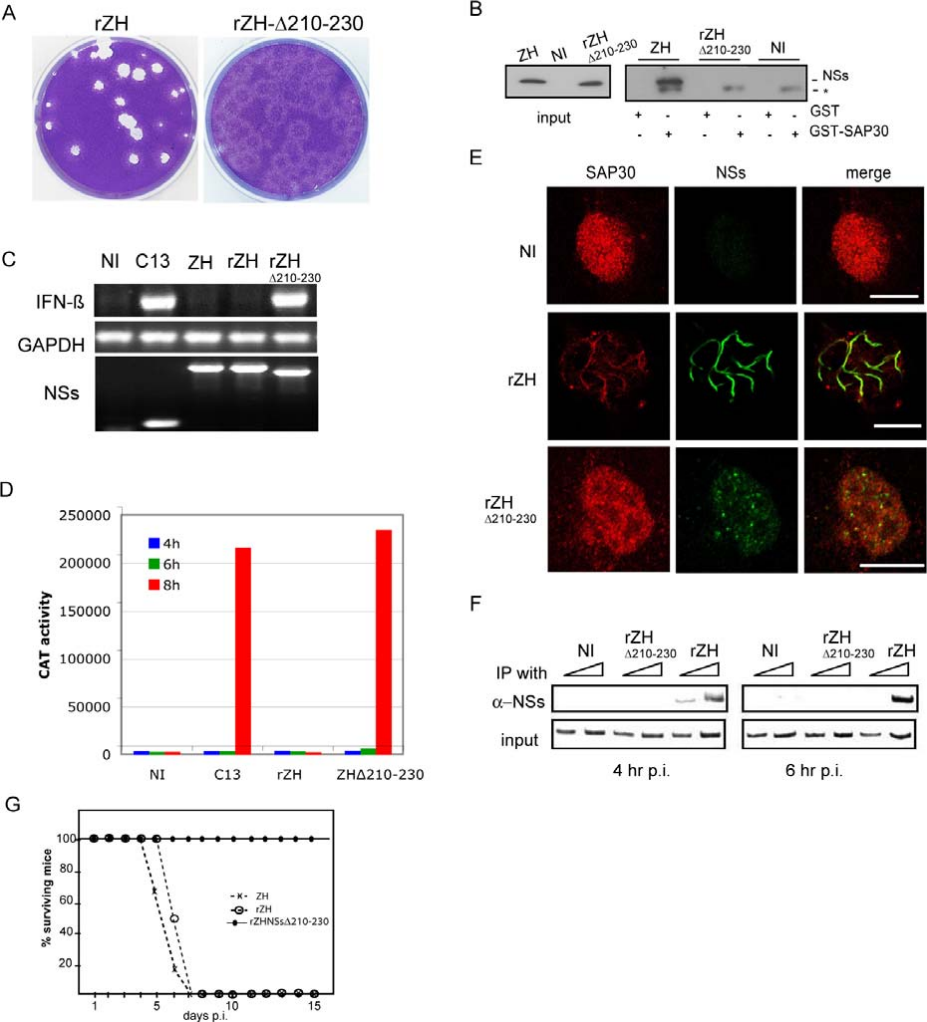
A Recombinant RVFV Which Does Not Interact with SAP30 Induces IFN-β and Is Avirulent (A) Monolayers of Vero cells infected with RVFV rec-ZH or rec-ZHΔ210–230 were fixed and stained with crystal violet at 5 days p.i. (B) GST or GST-SAP30 was incubated with extracts from cells uninfected or infected with rec-ZH or rec-ZHΔ210–230. Proteins from the crude extracts (input) or after binding on GST or GST-NSs beads were analyzed by Western blotting using anti NSs antibodies. * denotes a cellular protein bound on GST-NSs which copurified with NSs. BF (C) or L929 wt 330 cells (D, E, F) uninfected or infected with C13, wt ZH, rec-ZH, or Δ210–230 were incubated for 8 h (C), 18 h (E) or for the indicated time (D and F). Extracts were prepared and analyzed by RT-PCR to detect C) IFN-β, GAPDH, or NSs mRNA as described in [26]. (D) CAT activity, (E) confocal microscopy, or (F) chip experiment using anti-NSs antibodies like in Figure 3; (G) percentage of animals surviving after i.p. inoculation of 10^4^ pfu.

The region encompassing amino acids 210–230, comprised between two proline residues (which could possibly form a loop exposed for interactions) was found to be essential for SAP30 interaction (Figure 1D). To determine if there is a correlation between this interaction and the ability of the virus to inhibit IFN-β expression, we generated a recombinant RVFV encoding the mutated NSsΔ210–230 protein (rec-ZHΔ210–230) using the recently developed reverse genetics Pol I based-methodology (Billecocq et al, manuscript in preparation). The recombinant virus was successfully rescued with titers similar to wild type rec-ZH (approx 5x10^7^ pfu per ml at day 3 post transfection). Interestingly, the plaque morphology is different from the wt ZH or rec ZH (Figure 6A) and the viral genome was stable through several passages. Using the GST pull down assay, we demonstrated that in contrast with wt NSs, the mutated protein did not bind to GST-SAP30 (Figure 6B). To assess the ability of this mutated NSs to inhibit IFN-β, we infected murine BF or L929wt330 cells and analyzed the IFN-β expression, respectively, by RT-PCR (Figure 6C) or using the CAT reporter assay (Figure 6D). After infection with rec-ZHΔ210–230, expression of IFN-β was clearly detected in BF cells as well as in L929wt330 cells, whereas like the natural ZH, rec-ZH inhibited IFN-β expression. Using confocal immunofluorescence we observed that contrary to wild type NSs, nuclear NSs Δ210–230 protein did not form filaments and colocalized only very partially with SAP30 (Figure 6E). ChIP assays carried out after infection with the recombinant virus demonstrated that, unlike the wild type ZH NSs protein, NSsΔ210–230 protein did not interact with the IFN-β promoter (Figure 6F) whereas the recombinant wild type protein interacted with the promoter as early as 4 h p.i. as the natural ZH protein. In order to test pathogenicity of rec-ZHΔ210–230, 12 adult mice were inoculated with 10^4^ pfu via intraperitoneal route. All the animals inoculated with rec-ZHΔ210–230 survived, indicating that the mutant had completely lost its virulence, while all mice inoculated with rec-ZH or ZH died within 4–6 days. Overall, these results indicate that a.a. 210–230 of NSs are indeed essential for establishing an interaction with SAP30. They also indicate that the disruption of the NSs-SAP30 interaction abrogates the interaction of NSs with the IFN-β promoter, both these events being correlated with the incapacity of NSs to inhibit IFN-β expression and exert its pathogenic effect.

## Discussion

### After RVFV Infection a Multiprotein Complex Containing Viral NSs Protein and Host Factors YY1/SAP30/NCoR/Sin3A/HDAC-3 Is Recruited on the IFN-β Promoter

To evade the host antiviral response induced by IFNs, most viruses have evolved proteins that antagonize this response, targeting steps that are essential for triggering host innate immunity (for a recent review see [38]). Virulent ZH RVFV blocks the IFN-β gene expression of the host cell early after infection and by doing so, inhibits the host cellular antiviral response allowing the virus to pursue its infection through the organism. The previously described general inhibitory effect of NSs upon pol I and II-dependent transcription, which starts at 8 h after infection [25] could not be held as responsible for the inhibition of IFN-β gene expression that takes place at earlier times between 3–6 h after infection.

In this work, we demonstrate the existence of a novel mechanism induced early after ZH infection. It is based on the observation that SAP30, a subunit of transcription repressor complexes, directly interacts with the viral NSs protein, colocalizes with the NSs filament as early as 5 h p.i. and leads NSs to interact with the IFN-β promoter through transcription factor YY1 as soon as 4 h p.i. (Figure 3C).

**Figure 7:**
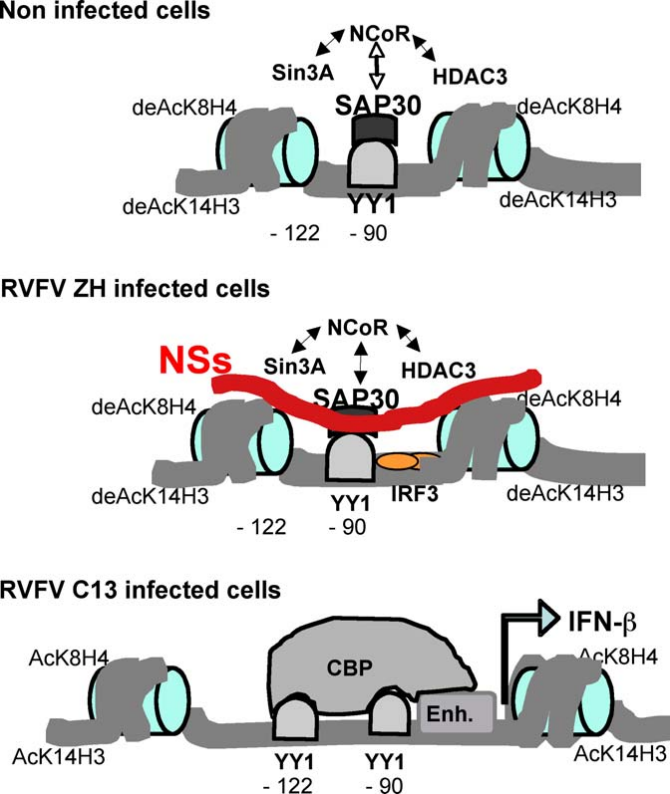
Schematic Representation of IFN-β Promoter during RVFV Infection In uninfected cells, a SAP30/Sin3A/NCoR/HDAC-3 corepressor complex, described here for the first time, to our knowledge, interacts with the constitutively repressed murine IFN-β promoter through its YY1 binding site present at position −90. After RVFV ZH infection, in the presence of NSs filaments, recruitment of corepressor complex SAP30/Sin3A/NCoR/HDAC-3 is reinforced whereas recruitment of co-activator CBP and of YY1 at its −122 site as well as acetylation of histone residues K8H4 and K14H3 is inhibited. Therefore, IFN-β promoter remains silent in spite of IRF3 nuclear translocation and binding to the promoter.

Using chromatin immunoprecipitation, we demonstrate here for the first time, to our knowledge, that during the constitutively silent state of the IFN-β gene, a complex containing SAP30/NCoR/HDAC3/Sin3A interacts with the IFN-β promoter. This interaction requires binding of YY1 at position −90, in agreement with previous results that identified this site as responsible for HDAC-dependent transcriptional inhibition of the IFN-β promoter [15]. This complex was released from the promoter after RVFV C13-induced promoter transcriptional activation but the situation was completely different in nuclei of ZH-infected cells containing the NSs filament where the promoter is maintained in a silent repressed state (Figure 7). In these cells, repression of the IFN-β expression occurred concomitantly with the stabilisation of the multiprotein complex containing NSs and YY1/SAP30/NCoR/Sin3A/HDAC-3 on the promoter region. Alongside with this recruitment, binding of YY1 to its −122 site and subsequent recruitment of CBP on the promoter region were strongly inhibited.

How is this multiprotein complex assembled? In the absence of structural data, we can only speculate based on the capacity of these different proteins to interact with each other. SAP30 is a subunit of repressor complexes containing corepressor Sin3A and/or NCoR and is also reported to directly interact with YY1. The interaction domains of SAP30 have been mapped: its N-terminal region interacts with NCoR and the C-terminal one with Sin3A [29] or YY1, the latter ones being mutually exclusive [31]. Sin3A has also been described to be able to directly interact with NCoR [39]. In the multiprotein YY1/SAP30/NCoR/Sin3A/HDAC3 complex, Sin3A is most probably directly interacting with NCoR rather than with SAP30 whose interaction with YY1 is required for the complex to be recruited on the promoter. The existence of two NCoR complexes has been reported, complex NCoR-1 containing SAP30/NCoR/HDAC3 and complex NCoR-2 containing SAP30/NCoR/Sin3A/HDACs 1, 2 and/or 3 [40,41]. Considering that HDAC-3 and Sin3A appeared to be enhanced on the promoter after ZH infection, while only traces of HDACs 1 and 2 were detected, this would suggest a preference for the presence of a NCoR-2 complex on the promoter.

In ZH infected cells, binding of YY1 to its −122 was inhibited whereas neither binding of YY1 to its −90 site nor IRF3 binding to the IFN-β promoter were affected. Therefore, the inability of YY1 to bind to its −122 site after ZH infection cannot be assigned to a general lack of accessibility of the promoter region. Of the two YY1 binding sites, the −122 site is the weaker one [16] and hence the most likely to be modulated. Noteworthy is that the YY1 −122 site is positioned on the NRDII region of the IFN-β promoter which is organized into a nucleosomal structure whereas the IRF3 site and the YY1 −90 site are positioned on the nucleosome free VRE region of the promoter. Therefore, enhanced histone deacetylation induced after ZH infection is expected to affect binding of YY1 to its −122 YY1 site more strongly than YY1 binding to its −90 site or IRF3 binding to the VRE.

### Could the NSs Filament Lead to the Formation of a New Silencing Compartment Inside the Nuclei of ZH-Infected Cells?

The relevance of the SAP30-NSs interaction for IFN-β inhibition and virus pathogenicity was clearly demonstrated by creating by reverse genetics a recombinant RVFV rec-ZHNSsΔ210–230, the NSs protein of which has lost its capacity to interact with SAP30. As a consequence, this virus was unable to form nuclear NSs filaments, did not inhibit IFN-β expression and was non pathogenic for the animal model. Even though we cannot exclude that the subnuclear redistribution of TFIIH components [25] contributes also to virus pathogenicity, the present data strongly suggest that the NSs-SAP30 interaction plays a determinant role for NSs filament formation, subnuclear redistribution and pathogenicity.

The disruption of the nuclear architecture caused by the filaments probably plays a role in maintaining IFN-ß gene in a repressed state. However, the repression is observed as early as 4 h p.i. a time when the filament is not yet formed and the nuclear organization not yet affected. In addition, our results clearly showed that NSs targeting the IFN-ß promoter requires an intact YY1 −90 site, suggesting that transcriptional repression does not merely result from NSs filament formation. Indeed, as shown in Figure 3D, NSs was unable to interact with the mut90 IFN-ß promoter mutated on its YY1 −90 binding site while the NSs filament was still formed.

Transcription factor YY1 as well as corepressors have the potential to interact either directly or indirectly with many promoter regions. This suggests that several regulatory DNA *loci* could be directed toward the NSs filament through these transcription factors and, by doing so, could be induced to a transcriptionally silent state. In this case, the NSs filaments would behave as a nuclear repressive compartment inducing silencing of particular genes. Theoretically, all the genes whose promoters interact with SAP30 and/or YY1 could be a target for NSs/SAP30-dependent abnormal transcriptional regulation, possibly explaining some of the pathogenic effects due the virus such as abortion, hemorrhagic fever, hepatitis or encephalitis. Further work will be necessary to address this issue.

## Materials and Methods

### Plasmids.

The cDNAs coding for NSs of Toscana and Germiston viruses were synthetised by RT-PCR from RNA extracted from virus infected Vero cells and cloned into BamHI site of pGBKT7 plasmid (Clontech) yielding pGBKT7-NSs_TOS_ and -NSs_GER_. The pGBKT7-NSs_ZH_ plasmid was described already [25]. The plasmid pCi-HA-SAP30 was constructed by inserting the HA-tagged full length murine SAP30 cassette from the pACT2 plasmid into the pCi vector at the BglII site (Promega). The SAP30 ORF sequence was also cloned into pCS2-Myc (kindly provided by A. Salic, Harvard Medical School) generating pCS2-mycSAP30 which expresses N terminal-c-myc tagged SAP30.

### Antibodies.

Rabbit polyclonal anti-NSs antibody (a generous gift from J. M. Egly, IGBMC, Strasbourg), raised against a peptide corresponding to the C-terminal 20 amino acids of the NSs protein was used for immunofluorescence experiments. Mouse anti-NSs polyclonal antibodies raised against the entire NSs protein [23] were used for Western blot, chromatin immunoprecipitations or immunofluorescence (Figure 5). Anti-IRF3 antibody used for chromatin immunoprecipitation was kindly provided by Michael David. Other primary antibodies used for immunofluorescence and chromatin immunoprecipitation experiments were: anti-SAP30 C-18 (sc-8471), anti-mSin3A AK-11 (sc-767), anti-NCoR C-20 (sc-1609), anti-CBP A-22 (sc-369), anti-YY1 H-10 (sc-7341) from Santa Cruz Biotechnology as well as anti-HDAC1 clone 2E10 (05–614), anti-HDAC2 clone 3F3 (05–814), anti-HDAC3 clone 3G8 (05–813), anti-AcK14H3 (06–911) and anti-K8H4 (06–760) from Upstate. Secondary antibodies used for immunofluorescence were Alexa 488 Chiken anti-goat (A21467), Alexa 555 Donkey anti-mouse (A31570) and Alexa 488 Chiken anti-rabbit (A 21441) from Molecular Probes. Antibodies used for immunofluorescence against the cellular proteins were checked by western blot for absence of cross-reaction with the NSs protein. The reciprocal experiment was also performed.

### Two hybrid system.

The two-hybrid screen was already described [25]. Briefly, a mouse cDNA library (CLONTECH) pretransformed in Y187 strain (MATα, trp1, leu2, *URA3*::(Gal1_UAS+TATA_)-*lacZ/MEL1*) was used for screening by mating with Saccharomyces cerevisiae AH109 strain (MATa, trp1, his3, ade2, leu2, *LYS2*::(Gal1_UAS+TATA_)-*HIS3*, *URA3*::(MEL1_UAS+TATA_)-*lacZ/MEL1*) transformed by pGBKT7-NSs_ZH_ . Yeast were grown in medium lacking tryptophan, leucine and histidine (SD medium) containing 5 mM 3-amino-1,2,4-triazole, a suppressor of unspecific *HIS3* expression. pACT-SAP30 plasmid expressing SAP30 from amino-acid 1–152 was rescued from colonies growing in the selecting medium and the insert was sequenced and analysed with the BLAST computer program. Interactions were assayed using the ß-galactosidase reporter or the ability to grow in selective medium.

### Viruses and cells.

Stocks of RVFV ZH548 and Clone 13 [32,33] were produced under BSL3 conditions by infecting Vero cells at m.o.i. of 10^−3^ and by harvesting the medium at 72 h p.i. Murine fibroblastic L929 cells and L929 wt330, mut90 and mut122 cell lines have been described previously [12,15]. Murine BF cells were already described [26].

### Rescue of ZH548 RVFV containing mutations in NSs.

The system to generation of infectious recombinant RVFV entirely from plasmids will be described in details elsewhere (Billecocq et al. manuscript in preparation). It is similar to those described [42,43] except for the use of pol I based plasmids [44] containing the L, M or S sequence of the ZH548 strain.

To introduce specific mutations in NSs we generated rec-ZH DelNSs in which the NSs coding sequence was replaced by a BbSI cloning site. We then used this plasmid linearized at the BbSI site to insert the mutated NSs ORF sequence which was amplified by PCR from pCi-NSsΔ210–230 generated by standard mutagenesis using specific oligonucleotides (available on request). The sequences were verified in order to ensure no unintentional mutations. The virus was rescued by transfecting BHK21-T7 cells (kindly provided by Dr Ito, Gifu, Japan), with the expression plasmids pTM1-L (0.5 μg) and pTM1-N (0.5 μg) together with 1 μg each of L, M and S Pol I plasmids. After 3 days, extensive cytopathic effect was normally observed, maintenance medium was collected and stored at −80°C. Working stocks were prepared by infecting Vero cells at moi of 10^−3^ as already described. This work was carried out in BSL3 conditions at the Pasteur Institute.

### CAT assay.

L929 wt330 cells seeded in six-well plates (200 000 cells/well) one day prior infection and grown in medium without G418 were infected with ZH548 and Clone 13 RVFV strains at a m.o.i of 5. The cells were harvested at different times after infection, and CAT activity was determined.

### GST pull down assay.

Plasmid pGex-4T-1 (Amersham Pharmacia) was used to express NSs or SAP30 fused in frame with the GST protein in E. coli. The GST-fusion protein purified on glutathione-sepharose beads (Amersham Pharmacia) was preincubated with BSA in Tris buffer (50 mM Tris HCl pH 7.5, 100 mM NaCl, 1 mM, 1 mM DTT, 0.05% Tween 20 and 1mM PMSF) and incubated with extracts of cells transfected with plasmid pCi-HA tagged-SAP30 in which murine SAP30 is expressed as a N-terminal HA-tagged protein or infected with ZH. After several washing with the same buffer containing 400 mM NaCl, the proteins bound to the beads were solubilized in Laemmli buffer and analysed by western blots using murine antibodies against GST, HA, or NSs.

### Coimmunoprecipitation experiments.

HEK 293 cells (approx. 8x10^6^) were transiently transfected by electroporation with 30 μg of the eukaryotic expression vector pCS2-Myc or pCS2-Myc-SAP30 which expresses SAP30 with c-myc at its N-terminus. After incubation for 24 h, cells were infected with ZH (m.o.i.=5). They were harvested at 24 h p.i and lysed in lysis buffer (1 % NP-40, 100 mM NaCl, 50 mM Tris HCl (pH 7.5), 1 mM EDTA, and protease inhibitor cocktail. Cell lysates were incubated overnight with protein G-Sepharose beads and anti-myc (9E10) antibody. Then, beads were washed with lysis buffer adjusted to 450 mM NaCl. Bound cellular proteins were separated by SDS-PAGE and subjected to western blotting using anti-myc-HRP (9E10), anti-NSs (rabbit polyclonal) antibodies.

### Chromatin immunoprecipitation.

Murine L929 cells or L929 wt330, mut90 and mut122 cell lines were infected by ZH or C13 RVFV strains and were fixed with 1% formaldehyde added to the medium for 10 min, scraped, and collected by centrifugation. Cells were resuspended in 300 μl ml of lysis buffer (5 mM piperazine-N,N′-bis(2-ethanosulfonic acid) (PIPES pH 8.0), 85 mM KCl, 0.5% NP-40) with a cocktail of protease inhibitors (Roche). Cells were pelleted by centrifugation and resuspended in 300 μl of 1% SDS, 10 mM EDTA, and 50 mM Tris-HCl (pH 8.0) containing protease inhibitors. After incubation on ice for 10 min, they were sonicated 6 times for 30 seconds using Bioruptor (Diagenode). Lysates were then cleared by centrifugation, and the concentration of DNA was determined. Equal amounts of DNA were diluted ten times in dilution-buffer (0.01% SDS, 1% Triton X-100, 1.2 mM EDTA, 16.7 mM Tris-HCl [pH 8.1], 167 mM NaCl). The chromatin solution was precleared for 45 min at 4°C on Protein A-Agarose/Salmon Sperm DNA beads from Upstate (16–157). After brief centrifugation and removing of the beads, DNA was incubated overnight at 4°C in a rotating wheel with 5 μl of the corresponding antibodies in 1000 μl final volume of dilution buffer. Immune complexes were collected on Protein A-Agarose/Salmon Sperm DNA beads from Upstate (16–157). Beads were washed sequentially in TSE (0.1% SDS, 1% Triton X-100, 2 mM EDTA, 20 mM Tris-HCl [pH 8.1]) with 150 mM NaCl, TSE with 500 mM NaCl, buffer A (0.25 M LiCl, 1% NP-40, 1% deoxycholate, 1 mM EDTA, 10 mM Tris-HCl [pH 8.1]), and one time with Tris-EDTA and then eluted with 200 μl 1% SDS and 0.1 M NaHCO3. Cross-links were reversed by heating at 65°C for 4 h after adding NaCl to 200 mM final concentration. After treatment with Proteinase K (50μg/ml for 1 h at 37°C), DNA was purified using Geneclean Turbo kit (Q-Biogene). PCR analysis of inputs or immunoprecipitated DNAs was performed as previously described [16]. Oligonucleotides F-40 and CAT, specific for the integrated muIFN-β promoters, were used as primers to amplify the integrated wt330, mut90 and mut122 promoters present in the corresponding cell lines and oligonucleotides 5.233 and 3.27 were used to amplify the endogenous wild-type murine IFN-β promoter present in L929 cells.

### Immunofluorescence and confocal microscopy analysis.

For immunofluorescence, ZH or C13-infected and uninfected L929 wt330 cells grown in twelve-well plates on coverslips were permeabilized with 0.5% Triton X-100 in 10 mM Pipes pH 6.8, 100 mM NaCl, 3 mM MgCl_2_ and 300 mM sucrose for 5 min at room temperature, fixed with 2% para-formaldehyde and incubated for 1h at 37°C with corresponding antibodies diluted in PBS/0.01% Tween/5 % BSA. Cells were then washed with PBS and incubated for 45 min at room temperature with corresponding secondary antibodies. Cells were observed with a Zeiss LSM 510 Axiovert 200 M microscope with confocal head. Images were collected in the z direction at 0.37 μm intervals. The images were analyzed by the LSM5 Image browser or Image J programs. Double-labeled pixels were displayed in yellow on the merge images.

### Animal experiments.

Lots of 12 four-week old mice (OF1, Charles River laboratories, France) were inoculated with 10^4^ pfu of RVFV via intraperitoneal route. Animals were observed twice daily for morbidity and mortality.

## Supporting Information

Figure S1 Localization of Endogenous N-CoR (A), and CBP (B) Proteins with NSs Filament Analyzed by Confocal Microscopy in L929 wt330 Cells (1.9 MB TIF)

Figure S2 Localization of HDAC1 (A), HDAC2 (B), and HDAC3 (C) Proteins with NSs Filament Analyzed by Confocal Microscopy in L929 wt330 Cells (3.1 MB TIF)

### Accession Numbers

The SwissProt accession number for the Sin3A associated protein 30 is 088574.

The EMBL-Bank accession number for the RVFV ZH548 NSs protein is DQ 380151.
