# Supplementary figures and images for: A SAP30 Complex Inhibits IFN-β Expression in Rift Valley Fever Virus Infected Cells
Source: PLoS Pathog. 2008 Jan 25;4(1):e13. doi: 10.1371/journal.ppat.0040013 (PMC2323286; doi:10.1371/journal.ppat.0040013)

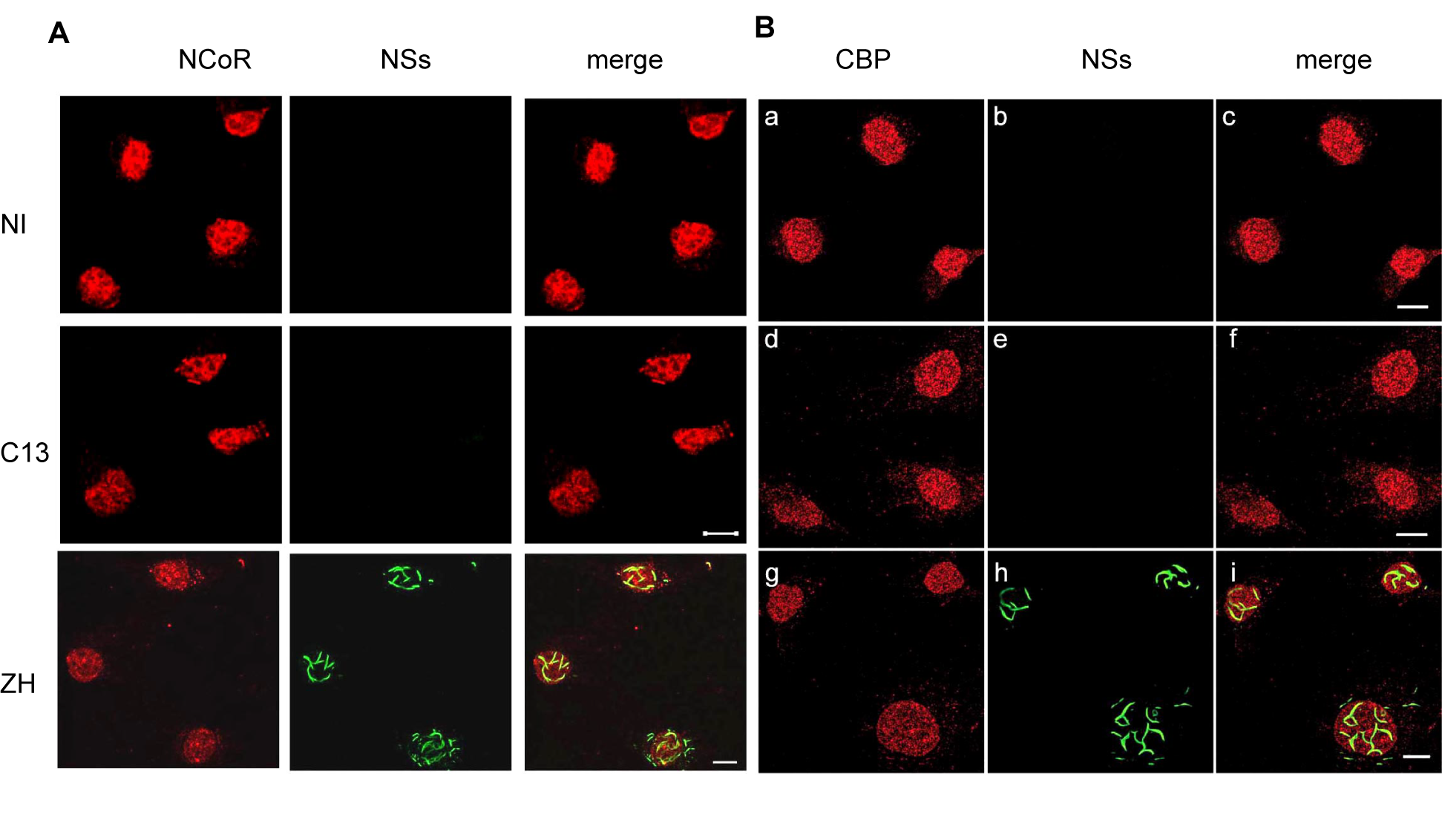

Supplement: Figure S1 — (1.9 MB TIF) [file ppat.0040013.sg001.tif]

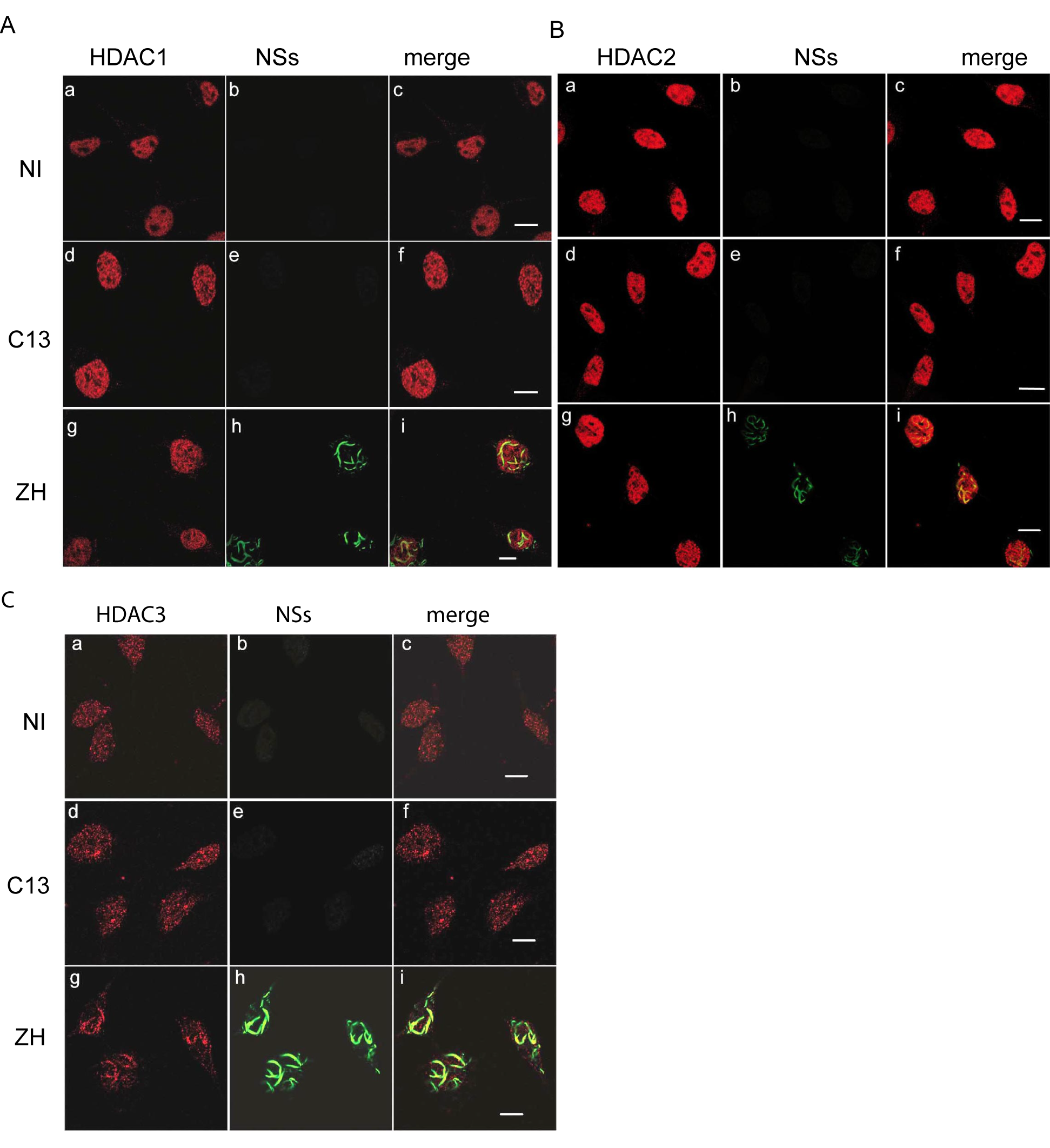

Supplement: Figure S2 — (3.1 MB TIF) [file ppat.0040013.sg002.tif]
